# Cyclic lipopeptides from *Bacillus amyloliquefaciens* D-1 control leaf spot in *Pseudostellaria heterophylla* via enhancing host immunity and inhibiting *Alternaria alternata*

**DOI:** 10.3389/fmicb.2026.1732585

**Published:** 2026-02-11

**Authors:** Haixia Shi, Shihua Zhou, Yanping Gao, Xue Jiang, Lang Qin, Lingling Wang, Xiaohong Ou, Yanhong Wang, Lanping Guo, Li Gu, Qing-Song Yuan, Tao Zhou

**Affiliations:** 1 Resource Institute for Chinese & Ethnic Materia Medica, Guizhou University of Traditional Chinese Medicine, Guiyang, China; 2 Guizhou Key Laboratory for Germplasm Innovation and Resource-Efficient Utilization of Dao-di Herbs, Guiyang, China; 3 School of Food and Biological Engineering, Jiangsu University, Zhenjiang, China; 4 State Key Laboratory for Quality Ensurance and Sustainable Use of Dao-di Herbs, National Resource Center for Chinese Materia Medica, China Academy of Chinese Medical Sciences, Beijing, China; 5 College of Bee Science and Biomedicine, Fujian Agriculture and Forestry University, Fuzhou, China

**Keywords:** *Alternaria alternata*, *Bacillus amyloliquefaciens* D-1, cyclic lipopeptides, leaf spot disease, *Pseudostellaria heterophylla*

## Abstract

**Introduction:**

*Pseudostellaria heterophylla*, a valuable medicinal herb in traditional Chinese medicine, is severely threatened by leaf spot disease caused by *Alternaria alternata*, leading to significant yield losses and mycotoxin contamination that compromises root quality and safety. This study aimed to characterize *Bacillus amyloliquefaciens* strain D-1 as a potential multifunctional biocontrol agent to mitigate this pathogen.

**Methods:**

Strain D-1 was isolated from the rhizosphere soil of *P. heterophylla*. Its antifungal efficacy was assessed through in vitro dual-culture assays and in planta pot experiments using both viable cells (Bc) and cell-free fermentation supernatant (Bf). Whole-genome sequencing was employed to identify biosynthetic gene clusters (BGCs), and high-performance liquid chromatography-mass spectrometry (HPLC-MS) was used to detect lipopeptides (LPs). Quantitative PCR and mycotoxin quantification were performed to evaluate the effects on fungal gene expression and toxin accumulation.

**Results:**

Strain D-1 demonstrated strong antagonistic activity, with Bf achieving a 54.7% inhibition rate against *A. alternata*. Genomic analysis revealed nine putative BGCs, including those for fengycin, iturin, and surfactin. HPLC-MS confirmed the production of these LPs, which effectively disrupted hyphal branching and spore germination. In pot trials, crude LP extracts reduced disease lesion area by 92.0% and suppressed key mycotoxin biosynthesis genes (PKSJ, PKSA, etc.), leading to a 77.0–98.1% reduction in toxin levels (ALT, AME, TeA, TEN). Additionally, strain D-1 and its LPs triggered systemic resistance in *P. heterophylla*, evidenced by elevated H₂O₂ accumulation and callose deposition.

**Discussion:**

The findings establish *B. amyloliquefaciens* D-1 as a novel biocontrol agent with triple mechanisms: direct antifungal action, suppression of mycotoxin biosynthesis, and induction of host systemic resistance. Its application offers a sustainable strategy to manage *A. alternata* in *P. heterophylla* cultivation, addressing both disease control and medicinal safety concerns. *heterophylla* cultivation, addressing both disease control and medicinal safety concerns.

## 1 Introduction

*Pseudostellaria heterophylla* (family Caryophyllaceae) is a commonly used traditional Chinese medicine and functional ingredient in Asia, with a historical of over 100 years (Qin et al., 2024; Yuan et al., 2024). Its tuberous roots contain a variety of bioactive components, including saponins, polysaccharides, amino acids, and trace elements (Hu et al., 2019), which exhibit anti-fatigue, anti-stress, immunomodulatory, and longevity-promoting effects (Hua et al., 2021). In recent years, escalating market demand of *P. heterophylla* has driven the expansion of cultivation areas and the adoption of prolonged monocropping practices, which have exacerbated the occurrence of various soil-borne and foliar diseases (Wang et al., 2023). Among these, leaf spot disease caused by *Alternaria alternata* is the most widespread, resulting in significant yield losses in *P. heterophylla* production (Wang et al., 2023). *A. alternata* also causes multiple diseases over 95% of plants either pre-harvest or post-harvest (Li et al., 2021), including leaf spot, fruit rot, or blight in major crops (e.g., tobacco, tomato, blueberry, cherry, and sunflower) and medicinal herbs (e.g., *P. heterophylla* and *Codonopsis pilosula*) (Shi et al., 2022). Additionally, *A. alternata* produces multiple mycotoxins, including alternariol (AOH), alternariol methyl ether (AME), altertoxin (ALT), alternic acid (AA), which possess genotoxic, hematotoxic, and carcinogenic activities (Louro et al., 2024; Yekeler et al., 2001).

For the management of diseases caused by *A. alternata*, conventional fungicides are the primary strategy for disease control but raise critical concerns about pathogen resistance, fungicide residues, toxicity to humans and animals, and environmental pollution (Lim et al., 2017; Steglińska et al., 2023). Developing resistant cultivars is a theoretically ideal eco-friendly strategy, yet it has limitations including lengthy breeding cycles and continuous germplasm renewal. Biological control, characterized by safety, environmental compatibility, and sustainability, is a core component of global organic agriculture strategies for managing pathogenic microbes during plant growth and storage (Wu et al., 2019; Zheng et al., 2019).

Notably, recent progress has been made in biocontrol strategies against Alternaria-derived diseases in both pre-harvest and post-harvest settings (Vasundaradevi et al., 2024). For instance, diverse bacterial isolates, including *Pseudomonas violacea* strain J-1, *Papiliotrema terrestris*, *Hanseniaspora uvarum*, *Rhodosporidium glutinis*, *Bacillus licheniformis*, and *B. pumilus*, have been isolated from various plants niches (e.g., soil, rhizosphere, and endosphere). These isolates exhibit significant inhibitory effects on *Alternaria* hyphal growth, thereby alleviating *Alternaria*-induced diseases such as kiwifruit soft rot (Li et al., 2021), blueberry soft rot (Li et al., 2024), tomato rot (Zahoor et al., 2022). Specifically, *Bacillus* spp., are a key group of biocontrol microbes, with distinct advantages in plant disease management due to their unique biological traits and pleiotropic antimicrobial mechanisms. These bacteria primarily suppress pathogen proliferation through competitive exclusion of ecological niches and nutrients, secretion of antimicrobial compounds (e.g., lipopeptide antibiotics, hydrolytic enzymes), and induction of systemic resistance (ISR) in plants (Santoyo et al., 2012). For instance, *B. amyloliquefaciens* strain S76-1 combats *Fusarium* head blight by secreting iturin A and plipastatin A, the lipopeptides that disrupts fungal cell membranes and inhibit fungal cell growth (Gong et al., 2015; Yuan et al., 2025). Even though, *Bacillus* spp. have been widely used to control various crop diseases (Sakiyo and Németh, 2023), there is still limited research on biocontrol strategies for managing of *Alternaria* derived diseases in Chinese herbal medicines.

In this study, a broad-spectrum antifungal strain D-1 was isolated from the rhizosphere soil of *P. heterophyllae* (Deng et al., 2023). Antifungal assays and *in vivo* biocontrol experiments verified the efficacy of D-1 against *A. alternata*-induced leaf spot disease in *P. heterophylla*. Genomic sequencing and liquid chromatography–tandem mass spectrometry (LC–MS/MS) were used to characterize the antimicrobial metabolites of D-1 as lipopeptides (LPs). Microscopic analysis revealed morphological changes in *A. alternata* hyphae and spores following treatment with D-1-secreted LPs. Systemic resistance responses induced by LPs were evaluated by analyzing hydrogen peroxide (H₂O₂) accumulation and callose deposition in *P. heterophylla*. Additionally, high-performance liquid chromatography (HPLC) and quantitative real-time polymerase chain reaction (qRT-PCR) were employed to assess *A. alternata* mycotoxin production in *P. heterophylla* tuberous roots. Collectively, *B. amyloliquefaciens* strain D-1 provides a novel resource for managing diseases in medicinal herbs and lays a foundation for sustainable development in the Chinese herbal medicine industry.

## 2 Materials and methods

### 2.1 Microorganisms, plants, and chemicals

*Bacillus amyloliquefaciens* strain D-1 was isolated via the dilution plating method from the rhizosphere soil of *Pseudostellaria heterophylla*, which was sampled in Shibing County, Guizhou Province, China (27°4′21”N, 108°8′0″E) (Deng et al., 2023; Yuan et al., 2024; Zhou et al., 2023) *Alternaria alternata* strain JK3-4, a causal pathogen of leaf spot disease, was isolated from symptomatic *P. heterophylla* leaves (Wang et al., 2023). For the evaluation of *in vivo* disease resistance, the *P. heterophylla* cultivar ‘Guishen 1#’ was used. Luria-Bertani (LB) agar medium and potato dextrose agar (PDA) medium was used to culture strain D-1 and strain JK3-4, respectively (Yuan et al., 2024; Zhou et al., 2023).

### 2.2 Antagonistic activity of strain D-1 against toxigenic pathogen *A. alternata*

Dual-culture antagonism assays were performed to assess the antifungal activity of strain D-1 and its metabolite extract against the toxigenic pathogen *A. alternata* strain JK3-4, following previously established protocols (Gong et al., 2019; Zhou et al., 2023). Briefly, a 10 μL aliquot of *A. alternata* strain JK3-4 conidial suspension (1.0 × 10^5^ CFU/mL) was inoculated at the center of potato dextrose agar (PDA) plates. Subsequently, to evaluate the antifungal efficacy of *B. amyloliquefaciens* strain D-1, its inhibitory activity against *A. alternata* was investigated by two independent methods. In the direct confrontation assay, a plug of the fungus was placed on a PDA plate, and 10 μL of bacterial suspension (OD₆₀₀ = 1.0) was spotted 3 cm away, with ddH₂O serving as a negative control. In the agar well diffusion assay, three wells were punched equidistantly (3 cm from the center) around a central fungal colony for the application of the test compound. Two wells were filled with 100 μL of metabolite extract, and a third well received 100 μL of methanol as a solvent control. The plates were then incubated, and the inhibition zones were measured. Each experiment was replicated three times. All plates were incubated in the dark at 28 °C for 5 days. After incubation, the radial growth of *A. alternata* strain JK3-4 was measured from the center of the conidial inoculation site to the edge of the mycelial colony. The antifungal inhibition rate was calculated using the following formula: Inhibition rate (%) = [(Control mycelial radius − Treated mycelial radius)/Control mycelial radius] × 100, where “Control mycelial radius” refers to the radial growth of *A. alternata* on ddH₂O-treated plates, and “Treated mycelial radius” refers to its radial growth on plates challenged with either *B. amyloliquefaciens* strain D-1 or its metabolite extract.

### 2.3 Genome sequencing of strain D-1 and prediction of its secondary metabolite biosynthesis gene clusters

The genome of *B. amyloliquefaciens* strain D-1 (GenBank ID in NCBI: PRJNA1333658) was sequenced using a hybrid approach combining PacBio RS II single-molecule real-time (SMRT) and Illumina sequencing, which was performed by Majorbio Bio-Pharm Technology Co., Ltd. (Shanghai, China). Genomic DNA libraries were prepared using the NEXTFLEX Rapid DNA-Seq Kit and sequenced on an Illumina NovaSeq 6,000 platform with paired-end reads (2 × 150 bp). Raw Illumina reads were processed using fastp (v0.23.0) to remove low-quality reads, reads containing excessive ambiguous bases (N), and short reads. Whole genome of strain D-1 were assembled using Unicycler (v0.4.8), followed by error correction employing the polished clean Illumina reads using Pilon (v1.22) (Wick et al., 2017).

Genome annotation was conducted using multiple gene prediction tools: Glimmer^1^ (Delcher et al., 2007), GeneMarkS (Besemer and Borodovsky, 2005), and Prodigal (Benson, 1999). Transfer RNA (tRNA) genes were identified with tRNAscan-SE (v2.0) (Chan and Lowe, 2019), and, ribosomal RNA (rRNA) were detected using Barrnap (ver. 0.9^2^) (Liu et al., 2019). Functional annotation of predicted genes was performed via homology-based searches using BLASTP, Diamond, and HMMER against the NR, Swiss-Prot, Pfam, GO, COG, KEGG, and CAZY databases. Secondary metabolite biosynthetic gene clusters were identified using antiSMASH (v5.1.2) with the MiBIG database.

### 2.4 Lipopeptide extraction and identification

Crude lipopeptides (LPs) were extracted using the acid precipitation method, as described in our previous study (Gong et al., 2015; Yuan et al., 2025). A single colony of *B. amyloliquefaciens* strain D-1 was inoculated into 20 mL of LB medium in a 100-mL Erlenmeyer flask, followed by incubation at 28 °C for 18 h. A 12-mL aliquot of the pre-culture was transferred to 200 mL of fresh LB medium in a 500-mL Erlenmeyer flask and incubated for 48 h under the same temperature conditions. The bacterial culture was centrifuged at 12,000 × g for 20 min at 4 °C to harvest the culture supernatant (cell-free fraction). The pH of the cell-free supernatant was adjusted to 2.0 using 6 M hydrochloric acid (HCl), and the mixture was stirred overnight at 4 °C to induce LP precipitation. The resulting precipitate was collected by centrifugation at 12,000 rpm for 10 min. The precipitate was washed three times with sterile ultrapure water, lyophilized (freeze-dried), and finally resuspended in methanol to obtain a crude LP solution. The identity of the extracted LPs was confirmed using reversed-phase high-performance liquid chromatography (RP-HPLC) and liquid chromatography-mass spectrometry (LC–MS), following the protocols established in our previous research (Gong et al., 2015).

Briefly, mass spectrometry analysis was conducted using an LC–MS-20 AD system (Shimadzu Corporation, Tokyo, Japan) equipped with a triple quadrupole mass analyzer and an ESI source operated in full positive scan mode. Chromatographic separation was performed using a C18 reversed-phase column (EXT-C18, 2.7 μm × 3.0 mm × 30 mm, packed with 5 μm particles) maintained at 40 °C. The flow rate was set to 0.2 mL/min. Mass spectrometry parameters included an ESI ion source operated in positive ion detection mode, a mass range of 100–1,500 Da, a detection voltage of 1.1 kV, a ion source voltage of 4.5 kV, and an ion source current of 6.5 μA.

### 2.5 Assessment of the biocontrol efficacy of *Bacillus amyloliquefaciens* strain D-1 and its metabolites against the leaf spot disease in *P. heterophylla*

The inoculants, comprising fresh cells of strain D-1 (Bc) with OD600 set at 1.0, its fermentation supernatant (Bf), and crude lipopeptides (LPs), were prepared as following our previously described method (Gong et al., 2015).

*P. heterophylla* seedlings at the eight-leaf stage were transplanted into pots (10 cm in diameter) and acclimated in a growth chamber under a photoperiod of 16 h light/8 h dark, with day/night temperatures maintained at 25/18 °C. 10 μL of an *A. alternata* spore suspension (1.0 × 10^5^ CFU·mL^−1^) was inoculated to the leaf center. At 2 days post-inoculation (dpi) with *A. alternata*, the seedlings were sprayed with Bc, Bf, or LPs; sterile double-distilled water (ddH₂O) was used as the control. All treatments were conducted under 90% relative humidity, with all seedlings maintained in the same growth chamber (under the aforementioned acclimation conditions). Each treatment included 8 independent seedlings. Disease severity was evaluated by lesion area method according to our previous study at 10 days post-treatment (dpt) with Bc, Bf, or LPs (Wang et al., 2023). The leaves lesion area in pathogen inoculation site was measured by ImageJ (version 1.53 t; National Institutes of Health, USA); each treatment calculated 12 leaves.

### 2.6 Inhibition of LPs on spore germination and mycelium

The assay was performed following the method described by Li et al. (2021). Briefly, 50 μL of a lipopeptide (LPs) solution (5 mg·mL^−1^) was added to 5 mL of potato dextrose broth (PDB) containing 1.0 × 10^5^ CFU·mL^−1^ spores of *A. alternata*. At 0, 4, 8, 12, 16, and 24 h post-inoculation (hpi), the optical density at 600 nm (OD₆₀₀) was measured using a Synergy 2 multimode microplate reader (BioTek Instruments, Winooski, VT, USA). Concurrently, the spore germination rate was determined by microscopic examination using an Olympus BX41 microscope (Olympus Corporation, Tokyo, Japan). After 5 days of incubation at 28 °C with shaking at 180 rpm, hyphal samples were collected, stained with calcofluor white (CFW), and observed under an Olympus IX73 fluorescence microscope (Olympus Corporation, Tokyo, Japan) to evaluate the treatment-induced morphological changes in *A. alternata* mycelia and spores.

### 2.7 Biocontrol of *A. alternata* and mycotoxin production in *P. heterophylla* tuberous roots by LPs

Twenty grams of *P. heterophylla* tuberous roots were placed in a sterilized sterile petri dish. The tuberous roots were then infected with 1 mL of *A. alternata* spore suspension (1.0 × 10^5^ CFU·mL^−1^) and treated with 1 mL of crude lipopeptide (5 mg·mL^−1^). Control samples were incubated with 1 mL of sterile methanol. All samples were incubated at 25 °C for 20 days, with three independent biological replicates per group. Photographs were taken for documentation, and all tuberous root samples were immediately stored at −80 °C for subsequent mycotoxin analysis.

### 2.8 Determination of mycotoxin production in *P. heterophylla* tuberous roots

Five major mycotoxins produced from *A. alternata* were analyzed using the PriboFastx® MFC311 Solid-Phase Extraction Column Test Kit (Pribolab, Qingdao, China), following the kit’s recommended protocols. The analytical workflow was as follows: Pre-dried *P. heterophylla* tuberous roots were ground into a fine powder. Exactly 2 g of the powdered sample was accurately weighed and transferred to a centrifuge tube. Ten mL of an acetonitrile/water solution (80:16, v/v) containing 0.1% (v/v) formic acid was added to the tube. The mixture was vigorously shaken for 30 min to facilitate mycotoxin extraction, then centrifuged at 6000 rpm for 5 min to separate the supernatant from the solid residue. Four mL of the resulting supernatants were carefully transferred to a new sterile tube and purified using a PriboFastx® MFC311 SPE column to remove interfering substances. The purified filtrate was dried via liquid nitrogen evaporation to concentrate on the mycotoxins. The dried residue was re-dissolved in 8 mL of methanol to form a mycotoxin-enriched solution. Two mL of the re-dissolved solution was passed through a 0.45-μm hydrophilic polyethersulfone (PES) filter to remove particulate matter, ensuring compatibility with subsequent chromatographic analysis. The filtered sample solution was subjected to mycotoxin quantification using a high-performance liquid chromatography (HPLC) system equipped with a photodiode array (PDA) detector (Shimadzu, Shanghai, China). For accurate quantitative analysis, analytical standards of the five target mycotoxins were purchased from Sigma-Aldrich (St. Louis, MO, USA): Alternariol (AOH), Alternariol monomethyl ether (AME), Altenuene (ALT), Tenuazonic acid (TeA), Tentoxin (TEN). These standards were used to generate calibration curves, which were applied to calculate the concentration of each mycotoxin in the *P. heterophylla* tuberous root samples.

### 2.9 Analysis of hydrogen peroxide (H_2_O_2_) accumulation and callose deposition in *P. heterophylla*

To detect hydrogen peroxide (H₂O₂) and callose, leaves of *P. heterophylla* treated with Bc, Bf, or LPs were harvested at 10 days post-treatment (dpt). H₂O₂ accumulation and callose deposition were visualized using previously described methods (Kumar et al., 2009; Millet et al., 2010; Yuan et al., 2025). Briefly, leaves were submerged in a 1 mg/mL solution of 3,3′-diaminobenzidine (DAB, pH 5.5) for 4 h, then boiled in 96% ethanol for 10 min to remove chlorophyll and stored in 96% ethanol. H₂O₂ accumulation was identified by the presence of reddish-brown staining in the treated leaves.

For callose detection, *P. heterophylla* leaves were fixed in a methanol: acetic acid solution (3:1, v/v) for 4 h. Following fixation, the leaves were sequentially dehydrated in 70% methanol for 2 h and in 50% methanol for an additional 2 h. After overnight hydration in deionized water, the leaves were washed three times with water and cleared by treatment with 10% (w/v) sodium hydroxide (NaOH) for 1–2 h. Following three further water washes, the leaves were stained in a solution containing 0.01% (w/v) aniline blue (Sigma-Aldrich, Shanghai, China) dissolved in 150 mM K₂HPO₄ (pH 9.5) for 3–4 h. Callose deposition was visualized using a Nikon H600L microscope under UV light illumination.

### 2.10 Mycotoxin biosynthesis gene expression analysis

To investigate the expression of toxin biosynthesis genes in *A. alternata*, mycelia of *A. alternata* treated with LPs were collected at 20 days post-treatment (dpt). Total RNA was isolated using the TransZol Up Plus RNA Kit (TransGen Biotech Co., Ltd., Beijing, China). RNA concentration and quality were assessed using a NanoDrop 2000 microspectrophotometer (Thermo Fisher Scientific, Wilmington, DE, USA) and by 1% agarose gel electrophoresis. First-strand cDNA synthesis was performed via reverse transcription using total RNA as the template and Anchored Oligo(dT)₁₈ primer (0.5 μg/μL), following the protocol provided with the EasyScript® One-Step gDNA Removal and cDNA Synthesis SuperMix Kit (TransGen Biotech Co., Ltd., Beijing, China). The synthesized cDNA was stored at −80 °C for downstream applications.

Six mycotoxin biosynthesis genes in *A. alternata*—including *TES1*, *TES*, *PksJ*, *PksA*, *PksI*, and *PksJF*—were selected for quantitative real-time PCR (RT-qPCR) analysis (Table 1). The primers used in this study were intron-spanning PCR primers. The *β-tubulin* gene was used as the endogenous reference gene for *A. alternata*. Relative gene expression levels were calculated using the 2^-ΔCT^ method.

**Table 1:** Primers used in RT-qPCR analysis.

Gene name	Forward primer	Reverse primer	Gene ID
*benA*	GTTGAGAACTCAGACGAGACCTTCTGCATTG	GAACCATGTTGACGGCCAACTTCCTC	MK558218.1
*PksJ*	GTCCCAAATTCCTACCCTCAC	GATAGCCATCGAAAGCATTCCC	ATN-PG05411
*PksA*	CGGTCTATCTCGTCCCTCAA	CGGCGTGCTGTAGTAGTGTAG	ATN-PG11581
*PksI*	TGGCATACGGGTAAGGTCTA	CGTGACGCTCTGGATAGTTC	ATN-PG01857
*PksJF*	CCTTCTTGTCGCCTACTTCAG	AACTCCATCGCATCCTCCAA	ATN-PG07778
*TES*	CTGATAGCCGAGCACTCCAG	TCGGATAAGGCGTTACGTCG	KT947104
*TES (1)*	CCGGACGACATGATGCAATG	ATCGATCCGTCGCTGTTCAA	KT947105

### 2.11 Statistical analyses

The results are presented as mean ± standard deviation. Differences were analyzed using Student’s *t*-tests and one-way analysis of variance (ANOVA) using Origin software (Version 2018, OriginLab Inc., Northampton, MA, USA).

## 3 Results

### 3.1 Strain D-1 exhibits antifungal activity against *A. alternata*

**Figure 1:**
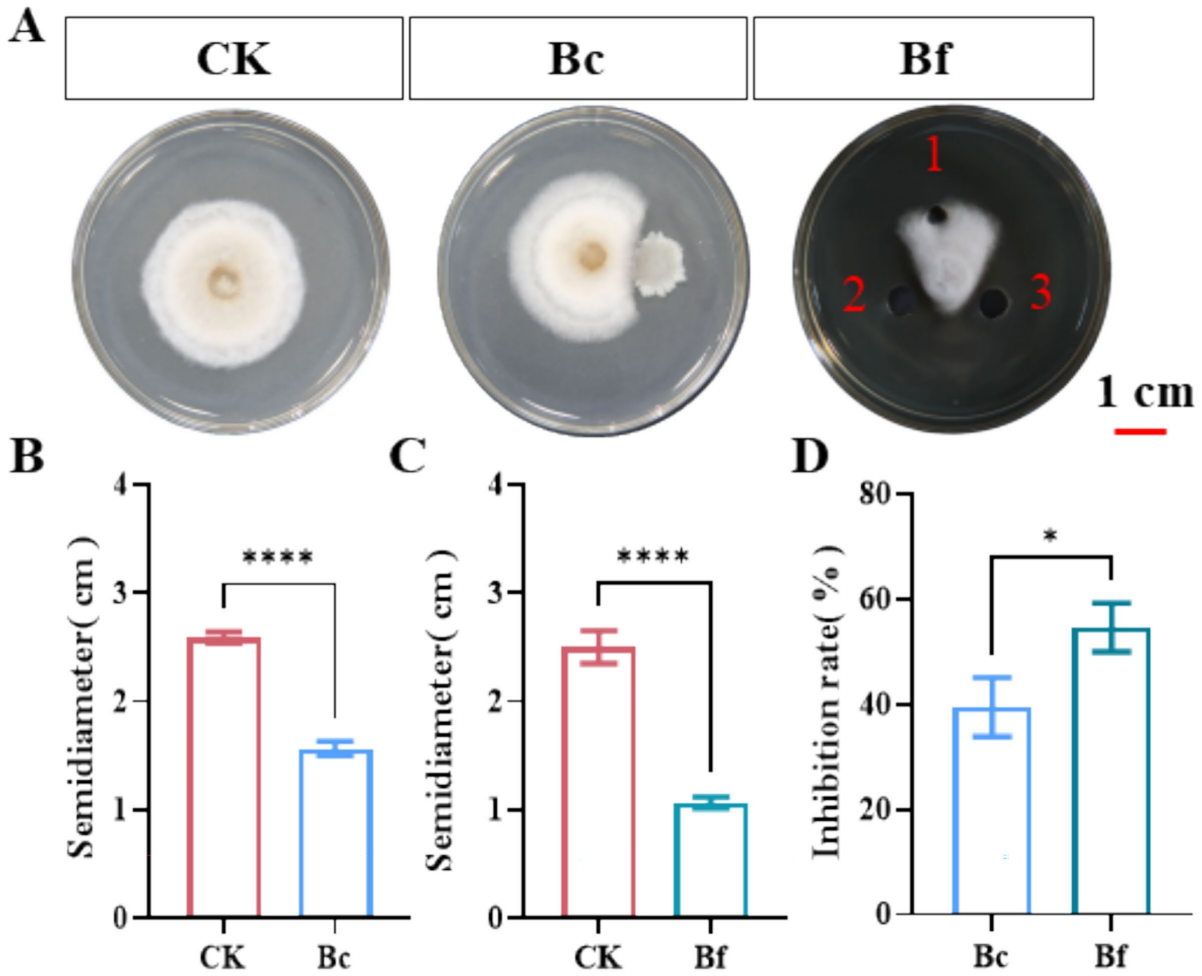
Inhibitory effects of *Bacillus amyloliquefaciens* strain D-1 (Bc) and its cell-free fermentation supernatant (Bf) on *Alternaria alternata*. **(A)** Colony growth inhibition of *A. alternata* at 5 days in the control (CK), Bc-treated, and Bf-treated groups. Scale bar = 1 cm. **(B)** Colony radius of *A. alternata* in the CK (distilled water) and Bc groups. **(C)** Colony radius of *A. alternata* in the CK (methanol) and Bf groups. **(D)** Inhibition rate of *A. alternata* in the Bc- and Bf-treated groups. CK, control group treated with distilled water and methanol; Bc, fresh cells of strain D-1; Bf, cell-free fermentation supernatant. One hole was methanol, 2 and 3 holes was cell-free fermentation supernatant. The bar charts show data from three biological replicates (*n* = 3). Values are presented as the mean ± standard deviation (SD). Statistical significance: *p ≤ 0.05, ****p ≤ 0.0001.

The antifungal activity of *B. amyloliquefaciens* strain D-1 was evaluated using dual-culture antagonism assays, as described in our previous studies (Gong et al., 2019; Zhou et al., 2023). Results showed that both live bacterial cells (Bc) and cell-free fermentation broth (Bf) significantly inhibited the growth of *A. alternata* (*p* < 0.0001) (Figure 1A). *A. alternata* colonies radius was significantly reduced in the presence of strain D-1 (1.6 cm) compared with the control (2.5 cm). Similarly, treatment with the cell-free fermentation supernatant of D-1 resulted in a smaller colony radius (1.1 cm) relative to the control (2.6 cm) (Figures 1B,C). Notably, the inhibition rate of the cell-free fermentation supernatant against *A. alternata* reached 54.7%, which was significantly higher than that of the live D-1 strain (39.4%) (Figure 1D). Collectively, these findings demonstrate that *B. amyloliquefaciens* D-1 effectively suppresses *A. alternata*, the causal pathogen of leaf spot disease in *P. heterophylla*, suggesting its potential as a promising biocontrol resource for managing this phytopathogen.

### 3.2 Genome assembly and antifungal metabolite biosynthesis gene clusters prediction of strain D-1

**Figure 2:**
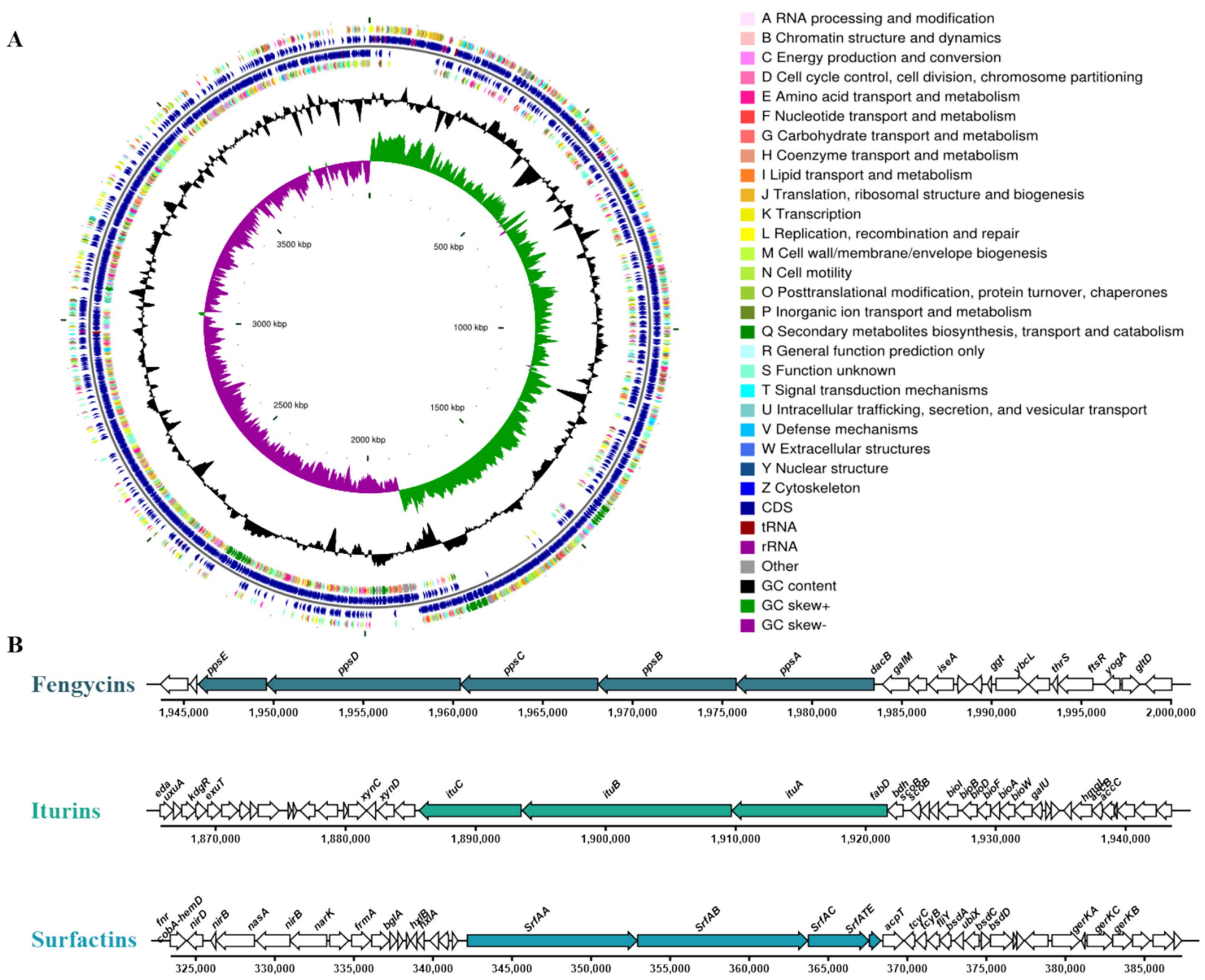
Genome map of *Bacillus amyloliquefaciens* strain D-1 and biosynthetic gene clusters of cyclic lipopeptides. **(A)** Circular genome map of strain D-1. Tracks from the outside to the center represent protein-coding genes colored by functional categories, gene distribution on the forward and reverse strands, genomic structural domains, GC content deviation, and GC skew. **(B)** Biosynthetic gene clusters (BGCs) of fengycins, iturins, and surfactins in strain D-1.

Genome sequencing analysis of *B. amyloliquefaciens* strain D-1 revealed a genome size of 3,980,777 bp with a GC content of 46.46%. The genome encodes 3,829 protein-coding genes (CDS), 86 tRNA genes, and 27 rRNA genes (Figure 2A and Table 2).

**Table 2:** Gene assembly and prediction of D-1 strain.

Item	D-1
Genome size	3,980,777
Number of total reads	3,522,567
GC content (%)	46.46
Number of CDSs	3,829
Number of rRNAs	27
Number of tRNAs	86
Number of sRNAs	81
Number of Chromo	1
Gene cluster	12
Genes assigned to NR	3,825
Genes assigned to GO	2,858
Genes assigned to KEGG	2,234
Genes assigned to COG	3,044
Genes assigned to Pfam	3,379

Prediction of secondary metabolite biosynthetic gene clusters (BGCs) in strain D-1 using antiSMASH (v5.1.2) identified at least nine putative biosynthetic pathways, including those for lipopeptides, polyketides, and terpenoids. Notably, the BGCs for fengycin, difficidin, bacillaene, bacillibactin, bacilysin, and macrolactin H showed 100% similarity to their respective reference clusters in the MiBIG database, while the surfactin biosynthetic cluster exhibited 82% similarity to its reference. These findings suggest that strain D-1 has high potential to synthesize these secondary metabolites, with detailed information on each BGC provided in Table 3. Collectively, genomic analysis highlights *B. amyloliquefaciens* D-1’s capacity to produce lipopeptides and polyketides—key classes of antifungal metabolites—thereby reinforcing its antifungal potential.

**Table 3:** The biosynthetic gene clusters of secondary metabolites in D-1 strain predicted by antiSMASH.

Similar cluster	Start (bp)	End (bp)	Similarity (%)
Fengycin	1,865,756	2,000,067	100
Difficidin	2,269,991	2,376,174	100
Bacillibactin	3,051,862	3,103,654	100
Bacilysin	3,639,963	3,681,382	100
Macrolactin H	1,384,085	1,471,921	100
Bacillaene	1,691,449	1,801,024	100
Surfactin	323,409	387,387	82
Butirosin A/B	924,056	965,301	7
Unknown terpenes	2,028,704	2,050,588	–

Further Clusters of Orthologous Groups (COG) functional annotation uncovered gene clusters encoding the lipopeptide biosynthetic machinery, including those for surfactins, iturin A, and plipastatin (fengycin B). The surfactin A biosynthetic cluster contains four core genes: *srfAA*, *srfAB*, *srfAC*, and *srfATE*. The iturin A biosynthetic cluster harbors three key genes: *ituA*, *ituB*, and *ituC*. Additionally, a distinct BGC associated with plipastatin biosynthesis was identified, including five key biosynthetic genes: *ppsA*, *ppsB*, *ppsC*, *ppsD*, and *ppsF* (Figure 2B). Collectively, these genomic insights confirm that *B. amyloliquefaciens* strain D-1 possesses the complete genetic blueprint for synthesizing lipopeptide antimicrobial compounds, further underscoring its potential as a robust biocontrol agent against phytopathogens.

### 3.3 Purification and identification of lipopeptides from strain D-1

**Figure 3:**
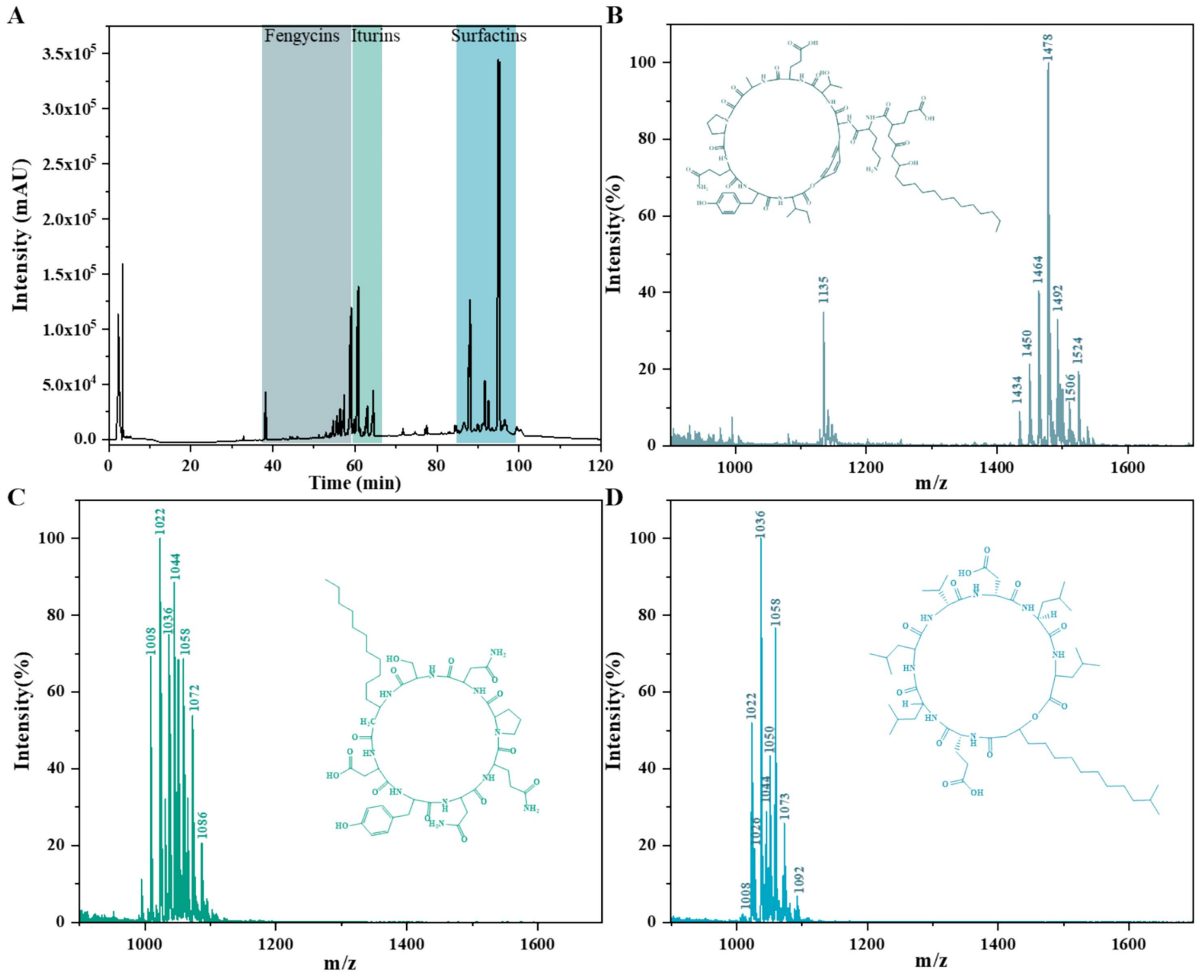
Identification of cyclic lipopeptides in *Bacillus amyloliquefaciens* strain D-1 using HPLC-MS. **(A)** Total ion chromatogram (TIC) of lipopeptides extracted from strain D-1, showing retention time ranges corresponding to fengycins, iturins, and surfactins. **(B–D)** Primary structures of representative members of the three lipopeptide families: fengycins **(B)**, iturins **(C)**, and surfactins **(D).**

HPLC analysis identified three major compound classes, with retention times for fengycins, iturins, and surfactins observed at 38.126–59.746 min, 60.327–64.860 min, and 86.829–96.593 min, respectively (Figure 3A). Based on subsequent characterization by HPLC–ESI-MS and comparison with references (Gong et al., 2015), the molecular weight spectra of the secondary metabolites were consistent with those of cyclic lipopeptides produced by Bacillus species, including fengycins, iturins, and surfactins. Molecular ion peaks [M + H]^+^ were detected at m/z 1,436, 1,449,1,461, 1,478, 1,491 and 1,506 for fengycins (Figure 3B); at m/z 1,044, 1,058, 1,073, and 1,086 for iturins (Figure 3C); and m/z 1,008, 1,022, 1,036, and 1,050 for surfactins (Figure 3D). Each compound class exhibited a 14 Da mass differences between consecutive homologs, indicating variation in fatty acid chain lengths within each group (CH₂ = 14 Da) (Table 4). Collectively, these findings confirm that strain D-1 synthesizes three major classes of cyclic lipopeptides: fengycin, iturin, and surfactin.

**Table 4:** The mass spectrometry of cyclic lipopeptides in strain D-1.

Compounds	Mass peaks(m/z)	Assignments
Fengycins (Plipastains)	1435.7[M + H]^+^	C14-FengycinA
	1449.9[M + H]^+^, 1471.8[M + Na]^+^	C15-FengycinA
	1461.9[M + H]^+^, 1483.8[M + Na]^+^	C16-FengycinA
	1478.1[M + H]^+^	C17-FengycinA
	1492.0[M + H]^+^	C16-FengycinB
	1506.2[M + H]^+^, 1528.1[M + Na]^+^	C17-FengycinB
Iturins	1044.1[M + H]^+^	C14-ItutinA
	1058.5[M + H]^+^	C15-ItutinA
	1073.1[M + H]^+^	C16-ItutinA
	1086.7[M + H]^+^	C17-ItutinA
Surfactins	1008.5[M + H]^+^, 1,031[M + Na]^+^, 1,046[M + K]^+^	C13-Surfactin
	1022.4[M + H]^+^	C14-Surfactin
	1036.5[M + H]^+^, 1,058[M + Na]^+^, 1,075[M + K]^+^	C15-Surfactin
	1050.0[M + H]^+^, 1072.7[M + Na]^+^, 1088.9[M + K]^+^	C16-Surfactin

### 3.4 Strain D-1 exhibits high biocontrol potential against the leaf spot disease of *P. heterophylla* caused by *A. alternata*

**Figure 4:**
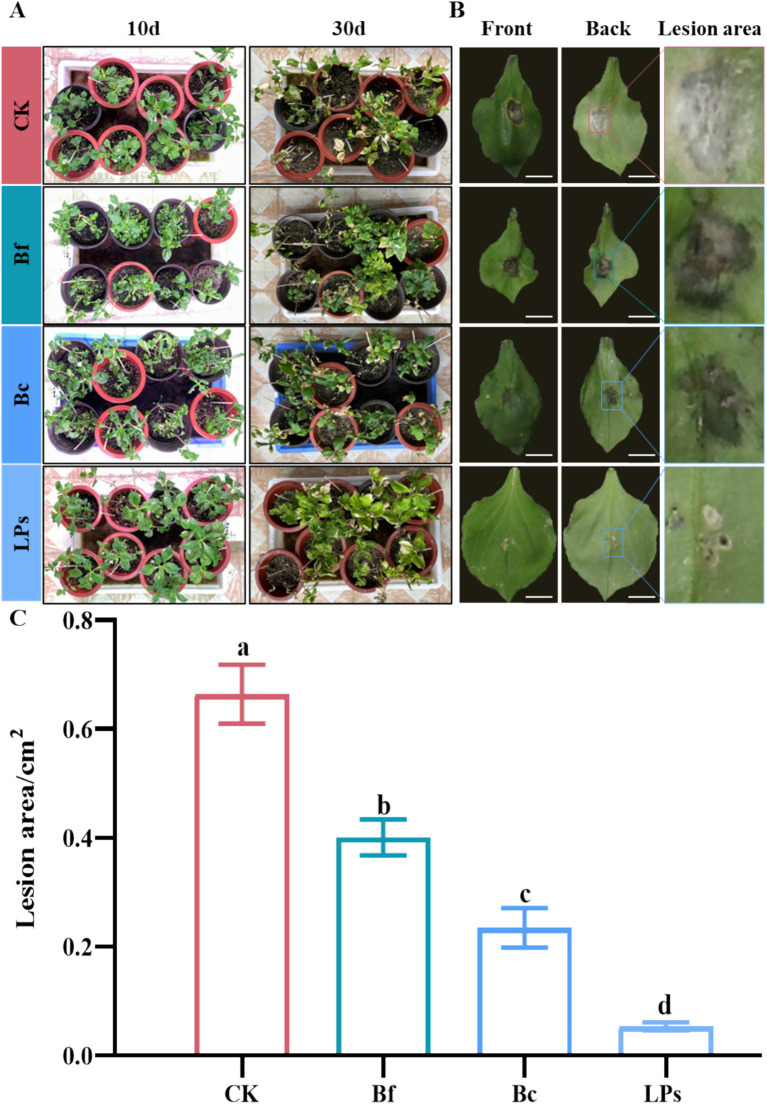
Biocontrol efficacy of *Bacillus amyloliquefaciens* strain D-1 and its metabolites against leaf spot disease in *Pseudostellaria heterophylla*. **(A)** Biocontrol efficacy of live cells (Bc), cell-free fermentation supernatant (Bf), and crude lipopeptides (LPs) against leaf spot disease in *P. heterophylla* at 10- and 30-days post-treatment. **(B)** Visualization of lesion areas on the adaxial and abaxial leaf surfaces of *P. heterophylla* in the Bc-, Bf-, and LP-treated groups. Scale bar = 1 cm. **(C)** Quantification of lesion areas at the inoculation sites on *P. heterophylla* leaves in the Bc-, Bf-, and LP-treated groups. Scale bar = 1 cm. Data are presented as the mean ± standard deviation from 12 biological replicates (*n* = 12). Different letters above bars indicate significant differences determined by one-way ANOVA followed by Tukey’s multiple comparisons test (*p* ≤ 0.0001).

A pot experiment was conducted to evaluate the biocontrol efficacy of Bc, Bf, and LPs. Foliar application of Bc, Bf, and LPs significantly suppressed the leaf spot development in *P. heterophylla* (Figure 4A). In contrast, water-treated control showed unrestricted mycelial colonization by *A. alternata* on the undersurfaces of leaves, with extensive hyphal growth observed (Figure 4B). Treatment with Bc or Bf restricted mycelial invasion, though slight dehydration was observed at pathogen inoculation sites. Notably, LP-treated leaves exhibited complete inhibition of surface mycelia, with no hyphal penetration detected on leaf surfaces (Figure 4B). The mean disease lesion area in the control group (0.66 cm^2^) was significantly larger than that in treatments with Bf (0.40 cm^2^), Bc (0.23 cm^2^), and LPs (0.05 cm^2^) groups (Figure 4C). Notably, LPs exhibited highest biocontrol activity against *A. alternata*, outperforming both Bc and Bf. These findings indicate that *B. amyloliquefaciens* strain D-1 and its metabolites, particularly lipopeptides, possess robust biocontrol potential against *A. alternata*-induced leaf spot disease in *P. heterophylla*.

### 3.5 Inhibitory effect of lipopeptides against hyphae growth and spore germination of *A. alternata*

**Figure 5:**
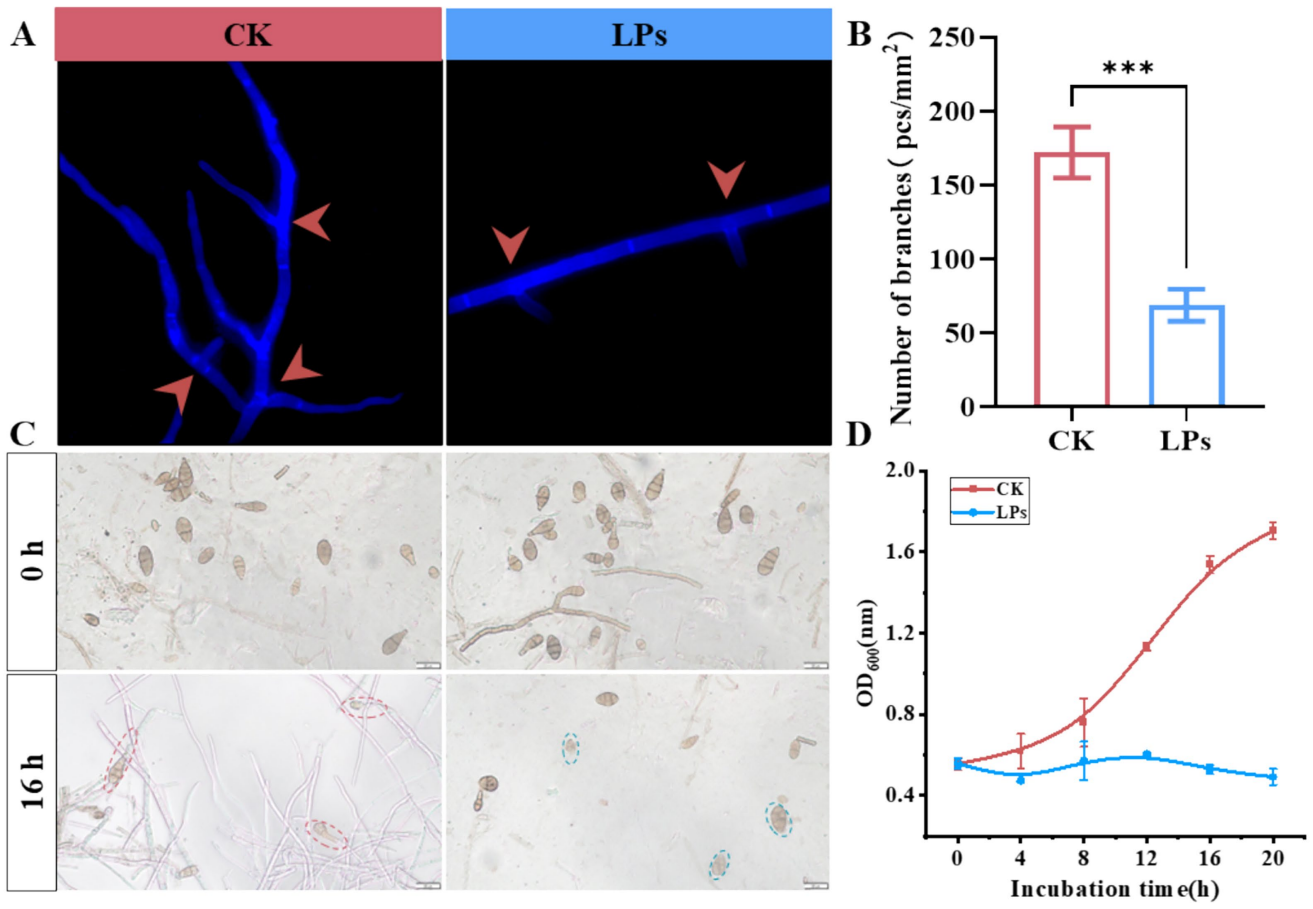
Inhibitory effects of cyclic lipopeptides (LPs) from *Bacillus amyloliquefaciens* strain D-1 on hyphal branching and spore germination of *Alternaria alternata*. **(A)** Representative fluorescence microscopy images showing hyphal branching (marked with red arrows) in the control (CK) and LP-treated groups. **(B)** Quantification of hyphal branch density (number of branches per mm^2^) in the CK and LP-treated groups. **(C)** Microscopic observation of *A. alternata* spore germination at 0 and 16 h in the CK and LP-treated groups. **(D)** Growth curve of *A. alternata* based on OD₆₀₀ values over a 20-h incubation period in the CK and LP-treated groups. Data are presented as the mean ± standard deviation (SD) from three biological replicates. Statistical significance: *** indicates significant differences at *p* ≤ 0.001.

Microscopic analysis revealed that treatment of *A. alternata* with crude lipopeptide (LPs) extract significantly inhibited mycelial development and branching (Figure 5A). Compared with the untreated control, LP-treated samples exhibited a 59.9% reduction in hyphal branch density, from 172 to 69 branches/mm^2^ (Figure 5B). Additionally, the antifungal effect on spore germination was assessed following treatment with crude LP extract, which resulted in significant suppression of germination (Figures 5C,D). Relative to the untreated control, nearly complete inhibition of spore germination was observed after 20 h of co-incubation in LPs-treated samples (Figures 5C,D). Collectively, these findings indicate that the lipopeptides inhibit *A. alternata* growth and development by disrupting hyphal branching and sporulation, thereby impeding fungal proliferation.

### 3.6 Inhibitory effect of lipopeptides against mycotoxins biosynthesis of *A. alternata*

**Figure 6:**
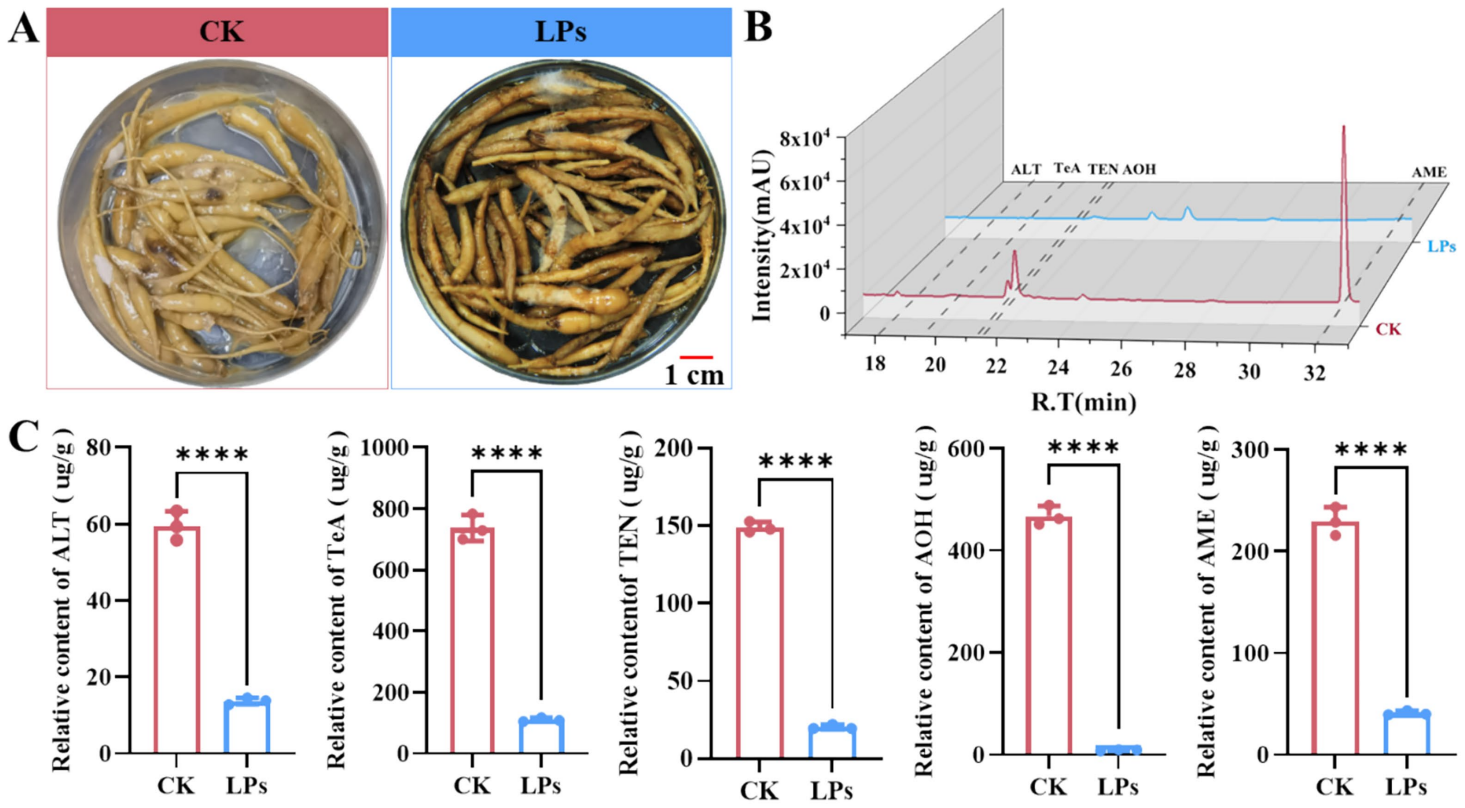
Inhibitory effect of cyclic lipopeptides (LPs) from *Bacillus amyloliquefaciens* strain D-1 on mycotoxin production by *Alternaria alternata* in *Pseudostellaria heterophylla* tuberous roots. **(A)** Visualization of *A. alternata* colonization in *P. heterophylla* tuberous roots after 20 days of co-incubation with LP treatment or sterile methanol (control, CK)**. (B,C)** Relative concentrations of mycotoxins alternariol (ALT), tenuazonic acid (TeA), tentoxin (TEN), altertoxin (AOH) and alternariol monomethyl ether (AME), in *P. heterophylla* tuberous roots co-incubated with *A. alternata*, quantified by HPLC-PDA (photodiode array detector). Data in bar charts are from three biological replicates (*n* = 3) and presented as the mean ± standard deviation (SD). Statistical significance: **** Indicates significant differences at *p* ≤ 0.0001.

**Figure 7:**
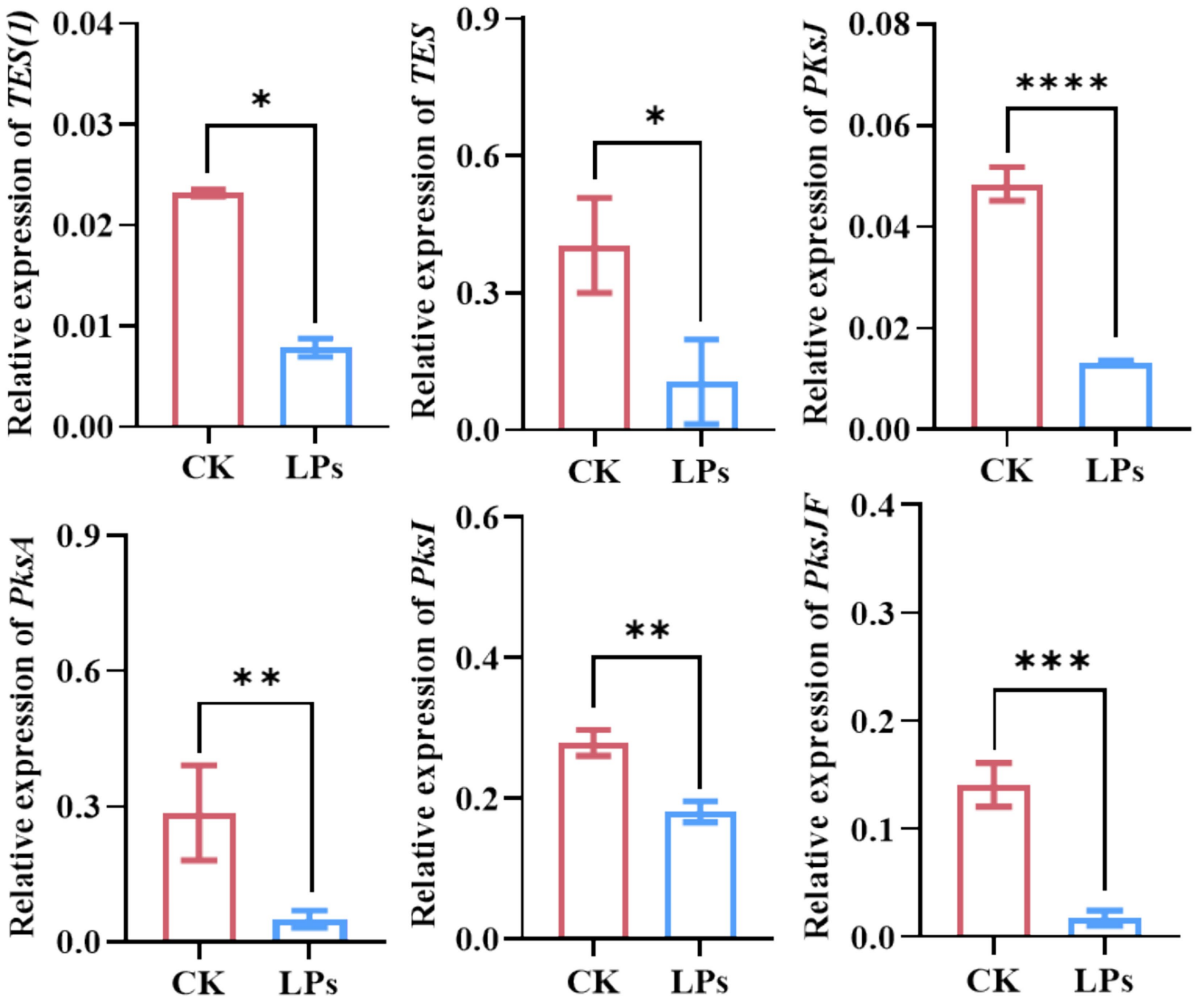
Relative expression levels of key mycotoxin biosynthesis genes in *Alternaria alternata* treated with cyclic lipopeptides (LPs). Data in the bar chart are from three biological replicates (*n* = 3) and presented as the mean ± standard deviation (SD). Statistical significance: *, **, ***, and **** indicate significant differences at *p* ≤ 0.05, *p* ≤ 0.01, *p* ≤ 0.001, and *p* ≤ 0.0001, respectively.

Following 20 days of treatment with LPs on *A. alternata*-infected *P. heterophylla* tuberous roots, infection assessments demonstrated significantly reduced *A. alternata* colonization compared to control samples, which exhibited dense mycelial growth (Figure 6A). HPLC-FAD analysis quantified mycotoxin levels in LPs-treated and control group, revealing significant reductions in the concentrations of alternariol (ALT, 13.70 μg/g vs. 59.5 μg/g), alternarol (AOH, 8.7 μg/g vs. 467.9 μg/g), alternaria monomethyl ether (AME, 41.0 μg/g vs. 229.4 μg/g), tenuazonic acid (TeA, 110.1 μg/g vs. 737.2 μg/g), and tentoxin (TEN, 20.3 μg/g vs. 148.8 μg/g) in LP-treated samples (Figures 6B,C). Previous studies have identified polyketide synthase (PKS) genes (*PKSJ*, *PKSA*, *PKSI*, and *PKSJF*) as key biosynthetic genes for alternariol (ALT), alternariol methyl ether (AME), and tenuazonic acid (TeA), while non-ribosomal peptide synthase (NRPS) genes (*TES* and *TES1*) were found to regulate TEN biosynthesis (Yun et al., 2015). Quantitative real-time PCR (qRT-PCR) analysis revealed that relative expression levels of *PKSJ*, *PKSA*, *PKSI*, *PKSJF*, *TES*, and *TES1* were significantly downregulated in treated samples compared to controls, showing reductions of 5.0-, 5.8-, 1.6-, 9.0-, 3.0-, and 2.5-fold, respectively (Figure 7). Collectively, these findings indicate that LPs from strain D-1 effectively suppress Alternaria mycotoxin biosynthesis—including alternariol (ALT), tenuazonic acid (TeA), tentoxin (TEN), alternariol monomethyl ether (AME), and altertoxin (AOH)—by regulating the expression of key biosynthesis genes.

### 3.7 Strain D-1 induced systemic resistance of *P. heterophylla* against the leaf spot pathogens

**Figure 8:**
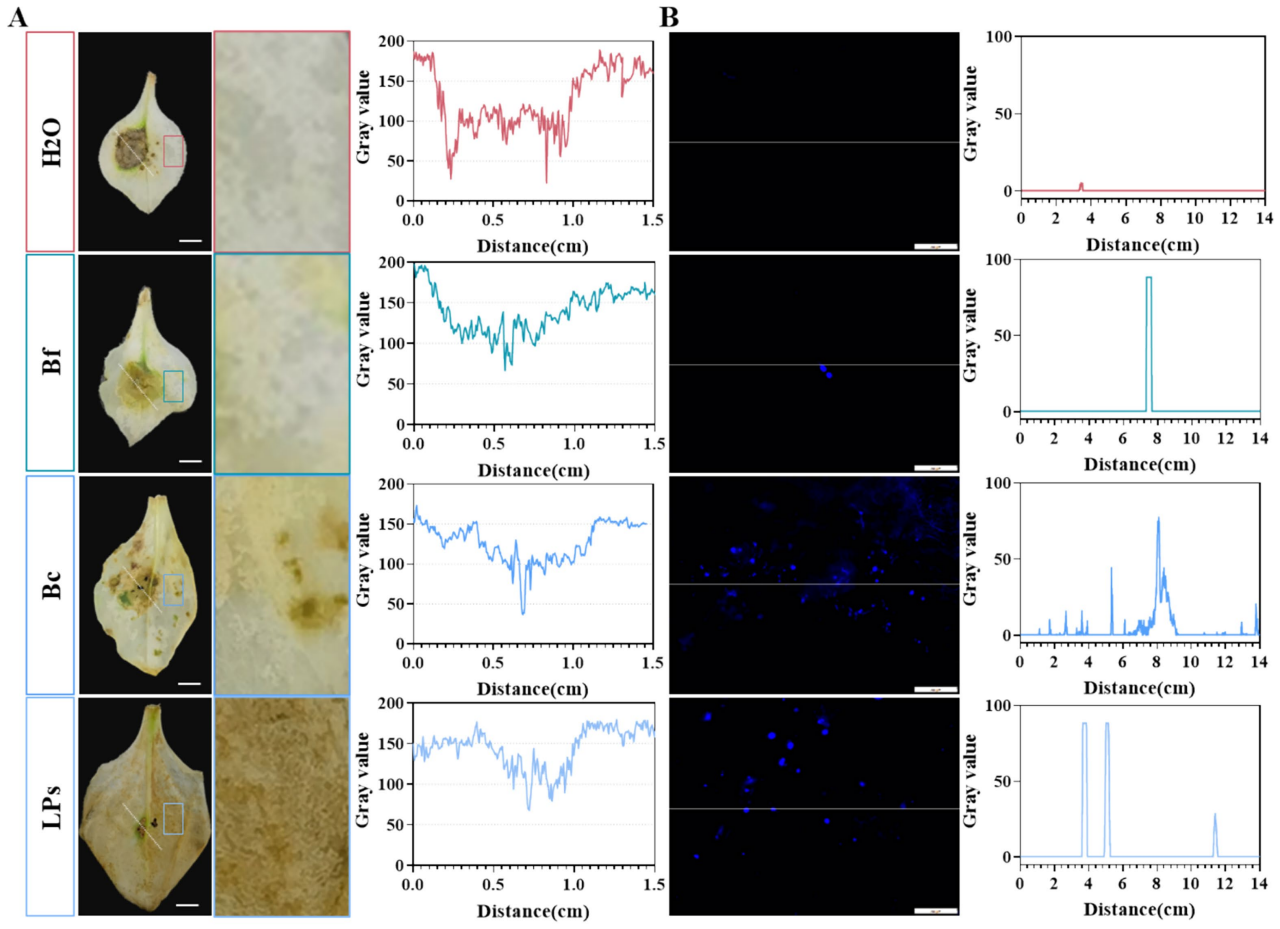
Induction of systemic resistance in *Pseudostellaria heterophylla* by *Bacillus amyloliquefaciens* strain D-1 and its cyclic lipopeptides (LPs) against leaf spot disease. **(A)** Accumulation of hydrogen peroxide (H_2_O_2_) in *P. heterophylla* leaves under different treatments, and quantification of lesion gray values. **(B)** Callose deposition in *P. heterophylla* leaves under different treatments, and quantification of fluorescence intensity. Scale bar = 1 cm.

H₂O₂ accumulation and callose deposition serve as a critical indicator of plants disease resistance (Wu et al., 2019). In this study, H₂O₂ accumulation (brown-yellow spots) were evaluated in *P. heterophylla* leaves treated with strain Bc, Bf, or LPs. Compared with control plants, reduced H₂O₂ accumulation was observed at the *A. alternata* inoculation sites in Bf-, Bc-, and LP-treated leaves (Figure 8A). Conversely, non-inoculated regions of treated leaves exhibited elevated H₂O₂ levels. Notably, Bc- and LP-treated leaves showed greater H₂O₂ accumulation than Bf-treated samples in non-inoculated regions.

Additionally, callose deposition (blue fluorescent deposits) was quantified in *P. heterophylla* leaves treated with Bc, Bf, or LPs. Relative to control plants, non-inoculated regions of treated leaves displayed increased callose accumulation. Similarly, Bc- and LP-treated leaves exhibited greater callose deposition than Bf-treated samples in non-inoculated regions (Figure 8B). These results suggest that strain D-1 and its lipopeptides can elicit a stronger oxidative stress response in planta against the pathogen, contributing to enhanced systemic resistance.

## 4 Discussion

### 4.1 Biocontrol potential of *Bacillus amyloliquefaciens* D-1 against *Alternaria alternata*

*Bacillus* species have emerged as pivotal biocontrol agents in agricultural systems, owing to their capacity to inhibit fungal pathogens and suppress disease development in crops, fruits, and vegetables (Bi et al., 2023; Salazar et al., 2023). These bacteria exhibit broad-spectrum antagonism against phytopathogenic fungi, including *A. brassicicola* (Wu et al., 2021), *Magnaporthe oryzae* (Li et al., 2018), *Botrytis cenerea* (Samaras et al., 2021; Yu et al., 2022), *Ralstonia solanacearum* (Yang et al., 2023), *Fusarium graminearium* (Gong et al., 2015), and *F. oxysporum* (Li et al., 2018). The present study demonstrates that *B. amyloliquefaciens strain* D-1 possesses significant potential for the biocontrol of *A. alternata*-induced disease*s*. The observed antifungal efficacy, particularly of the cell-free fermentation supernatant (Bf), is consistent with findings from other studies on *Bacillus* spp. (Zhao et al., 2022). These results highlight the potential of *B. amyloliquefaciens* strain D-1 as a novel biocontrol candidate for managing *A. alternata*-induced diseases in *P. heterophylla* and other economically important crops.

### 4.2 Genomic and metabolic insights into antifungal lipopeptide production

Genome mining of *B. amyloliquefaciens* D-1 revealed a rich potential for secondary metabolite synthesis, identifying nine putative biosynthetic gene clusters, including those for the key antifungal lipopeptides such as fengycin, iturin, and surfactin. The high sequence similarity (82–100%) of these clusters to reference genomes is consistent with findings in other biocontrol *Bacillus* strains, such as the model organism *B. velezensis* FZB42, which also possesses a similar repertoire of lipopeptide clusters crucial for its broad-spectrum antagonism (Chen et al., 2007). Notably, the surfactin cluster in strain D-1 exhibited a relatively lower identity (82%), suggesting potential structural variations. This result aligns with previous reports where the variations in lipopeptide synthetase gene cluster can lead to novel structural homologs with potentially enhancement bioactivity (Zeng et al., 2025). HPLC-MS analysis confirmed the production of these lipopeptide families, with mass spectra showing characteristic 14 Da intervals between homologs, corresponding to fatty acid chain length variations. This pattern of producing multiple lipopeptide homologs is a well-documented strategy in *Bacillus* spp., as demonstrated in *B. amyloliquefaciens* S76-3 (Gong et al., 2015), where the synergistic interaction between different lipopeptides resulted in superior antifungal efficacy. The ability of strain D-1 to co-produce these potent lipopeptides, particularly the high antifungal activity associated with iturin and fengycin families (Ongena and Jacques, 2008), provides a compelling molecular basis for its strong biocontrol performance observed *in vitro* and in planta against *A. alternata*.

### 4.3 Antifungal mechanisms of *B. amyloliquefaciens* D-1-derived lipopeptides

Previous studies have demonstrated that beneficial bacteria directly antagonize soil-borne phytopathogenic fungi and oomycetes by secreting antifungal compounds, including cyclic lipopeptides and volatile organic compounds (VOCs) (Wang et al., 2024). In this study, strain D-1 was found to directly inhibit *A. alternata* hyphal growth, branching, and spore germination through the production of cyclic lipopeptides. Cyclic lipopeptides from *Bacillus*, *Pseudomonas*, *Burkholderia*, and cyanobacteria exert their antimicrobial effects via strong amphiphilic properties that enable rapid insertion into fungal cell membranes, disrupting lipid bilayers and causing cytosolic leakage (Deleu et al., 2008; Gong et al., 2015; Gu et al., 2017; Melo et al., 2016). Specifically, iturin A and plipastatin A from *B. amyloliquefaciens* strain S76-3 induce severe morphological changes in *Fusarium graminearum* conidia and hyphae, including vacuolation, cytoplasmic leakage, and cellular inactivation (Gong et al., 2015). These findings suggest that strain D-1 disrupts *A. alternata* cell membranes through cyclic lipopeptides, thereby inhibiting fungal proliferation.

Additionally, previous studies have shown that *Bacillus* species can prime systemic resistance in plants, reducing disease incidence through activation of defense mechanisms (Xia et al., 2024). These responses include H₂O₂ accumulation and callose deposition, which are hallmarks of plants immune activation (Wu et al., 2019). In this study, strain D-1 induced significant systemic resistance in *P. heterophylla*, as evidenced by elevated H₂O₂ accumulation and callose deposition in non-inoculated leaf regions. Specifically, Bc- and LP-treated plants exhibited significantly higher H₂O₂ levels and callose deposition compared to Bf-treated and control plants. These findings suggest that both Bc and LPs contribute to systemic resistance induction, potentially acting synergistically to enhance plants immunity.

### 4.4 The potential function of *B. amyloliquefaciens* strain D-1 on mycotoxin management

Mycotoxin contamination, caused by phytopathogenic fungi such as *Fusarium* and *Alternaria*, represents a critical challenge to the safety of medicinal herbs and crops (Li et al., 2025). Beyond direct pathogen inhibition, an ideal biocontrol agent should also mitigate the impact of these toxic secondary metabolites. Our results indicate that lipopeptides from *B. amyloliquefaciens* D-1 effectively suppress the production of key *Alternaria mycotoxins,* including alternariol (AOH), alternariol monomethyl ether (AME), tenuazonic acid (TeA), and tentoxin (TEN). This suppression is likely achieved through the downregulation of core biosynthetic genes, a phenomenon consistent with recent findings that microbial antagonistic compounds can disrupt the transcriptional regulation of fungal secondary metabolism (Zhang et al., 2024). The ability of *Bacillus*-derived metabolites to interfere with mycotoxin biosynthesis pathways adds a crucial layer to their biocontrol profile, as preventing toxin accumulation is often more desirable than post-hoc removal (Yun et al., 2015).

Furthermore, certain *Bacillus* strains exhibit the capacity to degrade mycotoxins. This aligns with our observation of mycotoxin reduction and is supported by independent studies on strain D-1, which has been shown to biodegrade zearalenone into non-toxic metabolites (Deng et al., 2023). This dual capability, inhibiting biosynthesis and degrading existing toxins, is also reported in other biocontrol agents. For example, *B. velezensis* LS01 could simultaneously inhibit aflatoxin production and degrade aflatoxin B1 (Liu et al., 2023). The combination of these two mechanisms in the repertoire of strain D-1 highlights its significant potential as a multifunctional agent for comprehensive mycotoxin management in the production chain of *P. heterophylla*.

## 5 Conclusion

This study systematically evaluates *B. amyloliquefaciens* strain D-1 as a novel biocontrol agent against *A. alternata*-induced the leaf spot disease in *P. heterophylla*, a valuable medicinal plant. Through integrated phenotypic, genomic, and metabolomic analyses, the research provides mechanistic insights into its antifungal activities and highlights its multifunctional potential for sustainable disease management. Strain D-1 demonstrated strong inhibitory effects against *A. alternata* both *in vitro* and in planta. This efficacy was attributed to the production of cyclic lipopeptides (fengycin, iturin, surfactin), confirmed by HPLC-MS analysis, which inhibit hyphal branching and spore germination. Besides, cyclic lipopeptides can induce systemic resistance in *P. heterophylla* against the leaf spot disease. Additionally, LPs reduced *A. alternata*-derived mycotoxins (ALT, AME, TeA, and TEN) by 77.0–98.1% in infected *P. heterophylla* tuberous roots, concurrent with downregulation of their key biosynthesis genes (*PKSJ*, *PKSA*, *PKSI*, *PKSJF*, *TES*, and *TES1*). This research not only advances our understanding of *Bacillus*-fungal interactions but also paves the way for developing eco-friendly formulations to manage *Alternaria*-infected diseases in medicinal plants and crops.

## Data Availability

The datasets presented in this study can be found in online repositories. The names of the repository/repositories and accession number(s) can be found in the article/supplementary material.

## Footnotes

1
http://ccb.jhu.edu/software/glimmer/index.shtml

2
https://github.com/tseemann/barrnap
